# Reducing electronic media use in 2–3 year-old children: feasibility and efficacy of the Family@play pilot randomised controlled trial

**DOI:** 10.1186/s12889-015-2126-2

**Published:** 2015-08-14

**Authors:** Trina Hinkley, Dylan P. Cliff, Anthony D. Okely

**Affiliations:** Centre for Physical Activity and Nutrition Research, Deakin University, 221 Burwood Hwy, Burwood, Victoria 3125 Australia; Early Start Research Institute, Faculty of Social Sciences, University of Wollongong, Wollongong, New South Wales 2522 Australia

## Abstract

**Background:**

Participation in electronic media use among 2–3 year olds is high and associated with adverse health and developmental outcomes. This study sought to test the feasibility and potential efficacy of a family-based program to decrease electronic media (EM) use in 2–3-year-old children.

**Methods:**

*Family@play* was a six-session pilot randomised controlled trial delivered to parents of 2–3 year-old children from August to September 2012 in a community environment in the Illawarra region of New South Wales, Australia. Development of program content was guided by Social Cognitive and Family Systems Theories. The primary outcome was children’s electronic media use. Secondary outcomes included children’s time in sitting, standing and stepping. Data collectors were blinded to group allocation. Parents completed comprehensive process evaluation measures and participated in focus group discussions following completion of the program. Regression analyses were undertaken and effect sizes calculated using principles of intention to treat.

**Results:**

Twenty-two participants (*n* = 12 intervention; *n* = 10 control) provided complete baseline data; complete data from 16 participants (*n* = 6 intervention; *n* = 10 control) were available post-intervention. Process evaluation results were high, showing the acceptability of the program. Compared with children in the control group, there were greater decreases in total EM use among children in the intervention group (adjusted difference [95 % CI] = −31.2 mins/day [−71.0–8.6] Cohen’s *d* = 0.70). Differences for other outcomes were in the hypothesised direction and ranged from small for postural (sitting, standing, stepping) outcomes to moderate to large for individual electronic media (e.g. TV viewing, DVD/video viewing).

**Conclusions:**

This is the first family-based study to engage families of 2–3 year old children outside the United States and target multiple EM behaviours. *Family@play* was shown to be a feasible and acceptable intervention to deliver to families of 2–3 year old children. Potential efficacy is evident from moderate to large effect sizes. A larger trial is warranted to test the efficacy of the program.

**Trial registration:**

Australian New Zealand Clinical Trials Registry (ACTRN12612000470897).

## Background

Participation in high levels of electronic media (EM) use during early childhood (many studies use ≥2 h a day as a criteria based on the American Academy of Pediatrics’ recommendation [1]) has been linked with adverse health and social outcomes in young children, including increased weight status [2], behavioural problems [3], poorer language and cognitive development [4], and poorer social competence [5]. Current Australian and international recommendations are that children aged two-five years should engage in one hour or less of EM use (TV/DVD/video, computer use, electronic games) a day [1, 6, 7]. Nonetheless, participation in EM use is high during early childhood. Two-year-old children in the USA watch an average of two hours of television each day, with almost half (43 %) watching more than that amount [8]. However, those data do not include time spent in other types of EM use. Australian 2–4 year old children participate in 83 min per day of EM use, and only 26 % of those children meet the Australian recommendation [7, 9]. Further, young children spend up to 85 % of their day being sedentary [10–12]; that is, participating in behaviours in a sitting or lying posture while expending low levels of energy [13]. Such behaviours have been independently associated with adverse health outcomes even in children [14] and co-exist with multiple other unhealthy behaviours including poor dietary behaviours [15]. Such high levels of EM use and sedentary behaviours during early childhood are alarming as it is acknowledged that this period is foundational in the development of health behaviours such as these [16]. Further, television viewing and sedentary behaviours have been shown to track moderately during early childhood and childhood [17, 18]; therefore, once high levels of these behaviours are established, it is likely they will remain high. It appears, therefore, that by two years of age, these behaviours may already be established, high in prevalence, and stable.

As such, there is a need to test the feasibility, acceptability, and potential efficacy of interventions to promote appropriate levels of EM use in young children when EM behaviours are becoming established. A recent review [19] identified 13 studies which have previously attempted to reduce EM use during early childhood. However, all of those programs were undertaken in the United States and only one was a family-based program directly targeting parents of two or three year old children [20]. That study used only weekly newsletters or a single booklet to attempt to change health behaviours and targeted eating and physical activity behaviours; no effect was evident for children’s electronic media use. Given the majority of young children’s EM use occurs in the home environment, and that parents are key agents of change, there is a need to develop interventions to reduce EM use which target parents and young children in the home environment.

Research suggests that many parents do not perceive time in EM use as problematic, instead believing that it can be beneficial for their child [21]. Parents also perceive substantial challenges to reducing participation such as possible conflict within the family and lack of resources to support behaviours to replace EM use [22]. The *Family@play* intervention aimed to educate parents about the benefits of reducing EM use and provide them with support and strategies to minimise such behaviours in their children. The aims of this paper were to report on the feasibility and potential efficacy of the *Family@play* intervention.

## Methods

The study was a pilot randomised controlled trial. Assessors were blinded to participants’ group assignments. The study was performed and reported in accordance with the CONSORT statement [23]. The study was approved by University of Wollongong’s Human Research Ethics Committee. The trial was registered with the Australian New Zealand Clinical Trials Registry (ACTRN12612000470897).

### Setting and recruitment

The study was family-based, with weekly intervention sessions held at the University of Wollongong, New South Wales, Australia, central to the recruitment area. The study targeted families of two- and three-year-old children. Recruitment occurred through local community groups, such as playgroups, early childhood classes and childcare centres, where families with young children were likely to attend, and also with a University-wide email invitation. Recruitment was undertaken between March and July 2012. Written informed consent was obtained from all participant parents for themselves and on behalf of their child, and children’s verbal assent at time of data collection was obtained.

### Participants

Families were eligible to participate if they had a child aged two or three years at baseline. No other inclusion criteria were used. Exclusion criterion was diagnosis with a condition which may affect growth, development or behaviour such as Autism Spectrum Disorder. No families were excluded on these grounds. As this was a feasibility trial, no sample size power calculations were undertaken. Rather, this study aimed to recruit a sample of 20 participants to trial components of the intervention.

### Study design

The study was a pilot randomised controlled trial with assessments at pre- and post-intervention. Participants were enrolled by TH or a research assistant. Following baseline data collection, each participant was randomised to either the wait-list control or intervention group by ADO who created the randomisation sequence using a computer-generated random number producing algorithm in Microsoft Excel. Only ADO had access to the randomisation sequence (which was on a password-protected file). There were no restrictions on the randomisation. Participants were assigned to their groups by a research assistant.

The intervention was implemented over a five-week period from mid-August to mid-September following the baseline assessments. A trained facilitator, not part of the research team, delivered all program sessions. Participant families in the intervention group attended six, one-hour group sessions each week. Five of the sessions were targeted at both parents, with the sixth session focusing specifically on the role fathers play in their young children’s lives. Families in the wait-list control group attended the same sessions following the collection of all follow-up data. No changes to the trial methods were implemented after the commencement of the program.

### Theoretical underpinning

The program was theoretically informed, drawing on constructs from the Social Cognitive (SCT) [24] and Family Systems (FST) [22] theories. Within SCT, four key processes influence the learning, adoption, and maintenance of new behaviours: attention, retention, production and motivation. Those four processes, along with specific constructs from each of the three (personal, behavioural, environmental) levels of SCT, were embedded in the theoretical and practical components of the intervention, as depicted in Table 1 [24]. FST was used to better understand family dynamics and issues which may arise when change was introduced so they could be addressed within an anticipatory guidance approach [22].

**Table 1:** Session content and links with theoretical constructs

Session	Key content	Theoretical elements from SCT key content addresses		
		Personal level	Behavioural level	Environmental level
1	• Raise awareness of volume of child’s screen use & recommendations	• Knowledge	• Frequency	• Physical environment characteristics
	• Recognise benefits of reducing screen use	• Outcome expectancies	• Duration	• Social support/constraints
		• Motivation	• Mode	
		• Reinforcements/rewards	• Self-control/ regulation /goal setting	
			• Self-monitoring	
			• Behavioural capability	
2	• Correlates of young children’s EM use	• Reinforcements/rewards	• Frequency	• Social and/or physical environment
	• Strategies to spend less time in electronic media use – rules, say no, be active instead, have safe places in your home where your child can play on his/her own, encourage your child into other activities, decrease parent electronic media use, no TV in bedroom, no TV during meals, fewer TVs in home, use radio/CD for background noise	• Outcome expectations	• Duration	
		• Self-efficacy	• Mode	
		• Knowledge	• Self-monitoring	
		• Outcome expectancies	• Goal setting	
		• Motivation	• Self-control/ regulation	
			• Skills	
			• Role modelling/ observational learning	
3	• Neural networks and what happens in the brain when we try to change behaviour – change, resistance, reinforcement, new behaviour	• Reinforcements/rewards	• Frequency; or	• Social and/or physical environment
		• Outcome expectations	• Duration; or	
		• Self-efficacy	• Mode	
		• Knowledge	• Self-monitoring	
		• Outcome expectancies	• Goal setting	
		• Motivation	• Self-control	
			• Self-regulation	
4	• Behaviour change strategies – monitoring (and re-monitoring when necessary), planning (for normal and unusual days), goal setting (record goals, review), challenge identification & problem solving, praise and reinforcement	• Reinforcements/ rewards	• Frequency; or	• Social and/or physical environment
		• Outcome expectations	• Duration; or	
		• Self-efficacy	• Mode	
		• Knowledge	• Self-monitoring	
		• Motivation	• Goal setting	
	• Super family challenge – no electronic media for entertainment for the whole family for the whole week	• Emotional coping responses	• Skills	
			• Self-control/ regulation	
5	• Relapse prevention	• Reinforcements/rewards	• Frequency; or	• Social and/or physical environment
	• How to manage high risk situations	• Outcome expectations	• Duration; or	
	• Taking care of yourself	• Self-efficacy	• Mode	
		• Knowledge	• Self-monitoring	
		• Motivation	• Goal setting	
		• Outcome expectations	• Skills	
		• Emotional coping responses	• Behavioural capability	
		• Motivation	• Self-control/regulation	
		• Outcome expectancies		
		• Reinforcements/ rewards		
Fathers’ session	• Electronic media recommendations	• Reinforcements/rewards	• Frequency, duration and mode	• Social and/or physical environment
	• Benefits of reducing electronic media	• Outcome expectations	• Self-monitoring	
	• Importance of dads, dad-focused activities to do with child, especially Rough & Tumble play	• Self-efficacy	• Goal setting	
		• Knowledge	• Skills	
		• Motivation	• Behavioural capability	
		• Outcome expectancies	• Self-control/regulation	

### Intervention overview

The primary aim of the intervention was to decrease the total amount of time children spent using EM for entertainment. Sessions focused on three primary components:

- non-selectively decreasing EM use, for instance by use of budgeting and removing EM options from children’s bedrooms;
- selectively reducing EM use by setting rules/boundaries around use, and limiting participation based on context (for instance, only when child is not eating); and
- displacing EM use with other activities such as looking at books or being read to, imaginary games, independent play, etc.

Group sessions discussed the family-based activities to be undertaken by families and used an anticipatory guidance perspective to facilitate group-based problem solving to possible challenges. Each session included goal setting specific to each family’s circumstances and requirements. SMS messaging was used between group sessions to support adherence to goals: each participant received a personalised SMS encouraging them to achieve their previously-stated goal for the week. A healthy snack and childcare for children and siblings was provided during each of the group sessions. Content of each of the sessions is outlined in Table 1. The key messages of the program which were reinforced throughout the intervention were:

- Increase knowledge about EM recommendations and outcomes of EM use;
- Increase awareness and implementation of strategies to participate in healthy levels of EM use; and
- Teach families how to practice behaviour modification such as planning and monitoring.

### Measurement and data management

Data were collected in the families’ homes or at a location convenient to the family. At each data collection time point, parents completed a time-use diary (TUD) on each of four days and a survey, and children were weighed and measured by trained research assistants and fitted with an *activ*PAL™ accelerometer. Children wore the accelerometers during the same week that parents completed the TUD at both pre- and post-test. The *activ*PAL™ is a uni-axial accelerometer which classifies movement into sitting/lying, standing and stepping postures. It is a small (53 × 35 × 7 mm), lightweight (15 g) device which is worn on the mid-anterior aspect of the thigh. Baseline data were collected between July and August 2012 and follow-up data were collected between September and October 2012.

### Primary Outcome

#### Electronic media use

Detailed time children spent using electronic media was measured using a modified version of a TUD modelled on those with established reliability from a previous study [25]. TUDs were modified to include more EM options. Parents completed TUDs on three week days and one weekend day across the week during each period of data collection. In 15-min increments, parents reported the time their children spent using various types of electronic media including television, computer, active and sedentary electronic games and hand-held devices between the hours of 5 am and 11 pm daily. Data were reduced to mean minutes per day in each of the EM devices. Mean time in sedentary EM use (TV/DVD/video, computer, sedentary electronic games) and total EM use, as the primary outcome, were calculated.

### Secondary outcomes and covariates

#### Parent survey

Secondary EM use outcomes were derived from the individual EM behaviours in the TUD and time in sedentary EM behaviours. Parents completed a comprehensive survey. Items included in this study were: amount of time parent believed it was acceptable for child to use EM each day; weekly frequency child used EM to settle for bed; parental self-efficacy to support active opportunities (4 items); parent perception of the importance of participating in activities together (3 items); parental self-efficacy to limit EM use (11 items); parental beliefs about the influence of EM use on health, developmental and behavioural outcomes such as aggressive behaviour, attention, social skills (22 items); and the child’s opportunity to participate in EM use (17 items). Parents’ reported their responses on a five point Likert scale (strongly disagree to strongly agree). Responses for individual items were tested where appropriate for scale reliability. For items which had Cronbach’s alpha ≥0.7 [26, 27], scales were constructed by summing all responses and dividing by the number of items. The resultant scale scores were used in analyses.

#### Accelerometry

Time in sitting, standing and stepping postures was measured for one week using the *activ*PAL™ which has been validated in young children [28–30]. Each child was fitted with a monitor in a specially-made pouch on an elastic garter around the child’s right thigh. The monitor was positioned in the middle of the anterior aspect of the thigh and sewn into the material pouch before fitting. Elastic garters were attached by use of Velcro strips. Data were collected in 15 s epochs and reduced to percent of wear time in each of the postures. Children were asked to wear the monitors during all waking hours and remove them for sleeping and water-based activities (bathing, swimming). Each participant was required to have at least six hours of data on each of at least three week and one weekend days to be included in the analyses. Non-wear time was defined as 10 mins of consecutive zero counts and removed from daily wear time. Analyses were undertaken using percent of wear time in each of the postures to account for potential differences in wear time within and between participants.

### Process evaluation

Several process evaluation measures were used to assess the feasibility and acceptability of the program. These included attendance records, individual session evaluations, and end-of-program evaluations. Details of these evaluation measures, and general findings, are included in Table 2. Participant responses were on a 1–3 or 1–4 Likert Scale; facilitator responses were on a 1–5 Likert Scale.

**Table 2:** Individual session process evaluation items and results

Element of session evaluation	What this covered	Findings
Attendance records	• Facilitator recorded attendance	• Participants attended a mean of 78 % of sessions
Facilitator session evaluation	• How much of planned session delivered	• All content delivered as planned
	• Timing of session	• Sessions ran for mean of 68.6 mins (planned 60 mins)
	• Facilitator’s perspective:	
	○ content easy to understand	• 5.0/5
	○ content interesting; participants attentive	• 4.4/5
	○ participants engaged and contributed to group discussions	• 4.8/5
	• Quality of materials	• 4.5/5
	• Issues/concerns raised	• Decreasing ST when parents busy/tired/sick; spouse support/ motivation; boys boisterous and destructive
	• Gaps in content	• Discuss slow pace of behaviour change
	• Suggestions for improvement	• Include strategies for spouse support; address perceptions that Wii is good form of exercise; discuss how to manage low parent energy
Participant session evaluation	• Quality	• 3.2/4
	• Satisfaction	• 2.5/3
	• Usefulness	• 2.3/3
	• Relevance	• 2.3/3
	• Enjoyment	• 2.3/3
	• Learning opportunity	• 2.1/3
	• Facilitator’s knowledge, communication skills and approachability	• 2.4/3
End of program one hour semi-structured focus group	• Aspects of the program participants felt worked or could be improved	• Reported in process evaluation section of Results

### Potential efficacy

Potential efficacy was assessed by taking the following measures at baseline and post-intervention (between eight and 12 weeks after baseline), and comparing between-group changes in the following outcomes: EM use (time use diary), objectively measured sitting, standing and stepping time (*activ*PAL™ monitors) and parental characteristics such as self-efficacy for supporting healthy behaviours in their child and beliefs about risks and benefits of EM use.

### Analyses

All statistical analyses were undertaken in Stata 12.0 (Statacorp, TX). Descriptive statistics were calculated to describe the sample. Feasibility and acceptability were assessed using percentages and qualitative data as appropriate. Regression analyses controlling for group, baseline values of the outcome variable being assessed and a group x baseline EM use interaction (where an interaction was significantly associated with the outcome variable) were undertaken for each of the outcome variables. Analyses were conducted using both complete cases only and intention to treat (ITT) using baseline carried forward where necessary. As results were similar, and slightly more conservative for ITT, only ITT results are presented here. Due to the small sample size, centred values for baseline and follow-up variables were used to produce an unbiased estimate of the mean effect of treatment [31–34]. Effect sizes (Cohen’s *d* and Hedges *g*) were calculated. Cohen’s *d* was calculated by dividing the adjusted group mean difference by the error variance (root MSE from regression analyses) [31, 32, 35, 36]. Hedges *g* was calculated by adjusting Cohen’s *d* by control and intervention group sample sizes.

## Results

**Fig. 1:**
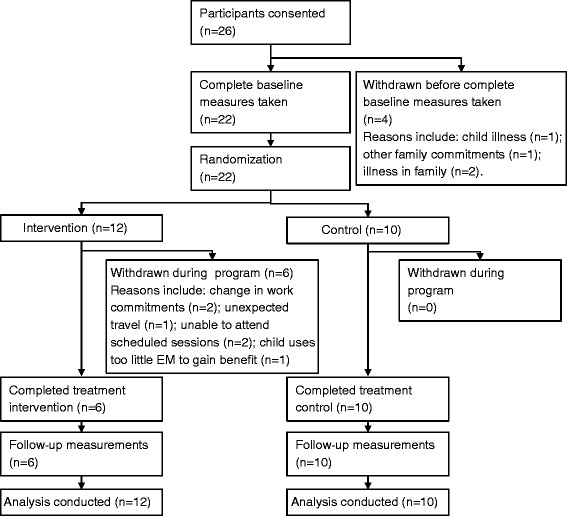
Flow of participants through the intervention

The flow of participants through the study is shown in Fig. 1. In total, 26 families initially consented to participate in the study; 22 provided complete baseline data and were randomised to intervention or control (see Fig. 1 for reasons for inability to provide baseline data). Following commencement of the program, 50 % of the intervention group withdrew (see Fig. 1 for reasons for withdrawal). Those completing did not differ on demographic variables such as age and sex, or the primary outcome of total EM use. However, completers were more likely to be highly educated and their children spent significantly more time in sedentary EM use at baseline (such as TV, DVD/video, computer use) than non-completers (*p* < 0.05 for both). The mean age of the children was 2.85 (±0.63) years for the control group (60 % boys) and 2.94 (±0.61) years for the intervention group (67 % boys) at baseline. All respondents were the mother of the child in the study and had a mean age of 34.85 (±3.13) years in the control group and 32.37 (±9.7) years in the intervention group (*p* > 0.05). All participants in the control group and 75 % of participants in the intervention group were born in Australia, with the remainder being born in the United Kingdom or Ireland. All participants spoke English as their primary language at home.

### Process evaluation

Recruitment targets were achieved. Results for each of the items in the weekly process evaluation are presented in Table 2. All sessions were implemented and all content within each session was delivered as planned.

Results of the end of programme focus groups showed that participants generally enjoyed the program and found it beneficial both for themselves and their child. For instance, “I really liked the ideas for different activities … I think that’s why they were getting bored and nagging to watch TV because I was just doing the same things every day … and I found that (the activities) really beneficial”; “The brain networks (session) really concreted the fact that we still can change it (behaviour)”; “My children sleep better – they go to bed earlier and more easily and sleep for longer without TV before they go to bed”; “The weekly challenges were very useful. sort of showed you can change it in little tweaks, little ways and it doesn’t have to be a big thing … and you can tailor it to what you need.”

In addition, participants identified some aspects of the program they thought could be improved. These included the following: sharing activities other families used to entertain their children, having fewer face-to-face sessions and some online content they could access at their leisure and when needed, and one parent reported that her child suffered night terrors following removal of TV viewing before bed but added that these only lasted a few nights before abating.

### Potential efficacy

Table 3 shows the mean time children spent in each of the EM behaviours at baseline and post-intervention. Children in the intervention group reduced their total EM use by 39 mins, while children in the control group increased their total EM use by 3 mins, resulting in a moderate effect (*d* = 0.70). Sedentary EM use was reduced by 36.3 mins for the intervention group and 2.6 mins for the control group (*d* = 0.57). Several individual EM behavioural reductions also resulted in moderate (≥0.5) to large (≥0.8) [37] effect sizes, including DVD/video viewing and handheld e-game use.

**Table 3:** Time in EM use at baseline and post-intervention, adjusted difference and effect sizes for electronic media use outcomes

Outcome measure (mins/day)	Baseline (*M,* 95 % CI)		Post-intervention (*M,* 95 % CI)		Adjusted difference^a^ (*M,* 95 % CI)	Effect Size (Cohen’s d)^b^	Bias corrected (Hedges g)
	Control	Intervention	Control	Intervention			
Total EM use	97.1 (61.5, 132.8)	117.8 (74.0, 161.6)	100.1 (64.1, 136.1)	78.8 (44.7, 112.8)	−31.2 (−71.0, 8.6)	0.70	0.39
TV/DVD/Video/computer/ sed e-games combined	90.0 (56.9, 123.1)	104.4 (60.3, 148.4)	87.4 (50.5, 124.3)	68.1 (38.6, 97.7)	−24.5 (−62.9, 13.8)	0.57	0.32
Television viewing	78.4 (45.5, 111.3)	60.0 (26.0, 94.0)	74.6 (43.6, 106.7)	49.7 (21.3, 78.1)	−16.1 (−50.5, 18.4)	0.42	0.23
DVD/Video viewing	7.1 (−4.1, 18.4)	44.4 (13.5, 75.3)	7.5 (−4.9, 19.9)	18.4 (−2.7, 39.5)	−14.2 (−34.1, 5.6)	0.64	0.36
Educational computer use	3.8 (−1.3, 8.8)	0.0 (0.0, 0.0)	5.3 (−2.7, 13.2)	0.0 (0.0, 0.0)	−0.5 (−4.4, 3.5)	0.10	0.06
Computer for entertainment	0.8 (−0.9, 2.4)	0.0 (0.0, 0.0)	0.0 (0.0, 0.0)	0.0 (0.0, 0.0)	0.0 (0.0, 0.0)	-	-
Sedentary e-games (e.g. PlayStation)	0.0 (0.0, 0.0)	0.0 (0.0, 0.0)	0.0 (0.0, 0.0)	0.0 (0.0, 0.0)	0.0 (0.0, 0.0)	-	-
Active e-games (e.g. Wii)	0.0 (0.0, 0.0)	0.0 (0.0, 0.0)	3.8 (−4.7, 12.2)	0.0 (0.0, 0.0)	−3.8 (−10.9, 3.4)	0.47	0.27
Digital tablet	4.5 (−1.7, 10.7)	8.1 (−5.6, 21.8)	4.1 (−1.3, 9.6)	6.3 (−7.5, 20.0)	−1.0 (−8.4, 6.3)	0.13	0.07
Handheld e-game	2.6 (−2.4, 7.7)	4.1 (−1.6, 9.8)	4.9 (−4.4, 14.2)	3.1 (−2.4, 8.7)	−3.7 (−6.8, −0.7)	1.09	0.62
Electronic toy	0.0 (0.0, 0.0)	1.3 (−0.86, 3.4)	0.0 (0.0, 0.0)	1.3 (−8.6, 3.4)	0.0 (0.0, 0.0)	-	-

^a^Adjusted for group and baseline value of outcome variable

^b^Effect sizes calculated using centred means of baseline carried forward values where data were missing

Table 4 shows the changes in potential mediators and *activ*PAL variables from baseline to post-intervention for the intervention and control groups. A moderate effect size was evident for parental beliefs about the influence of EM use on their child’s health, developmental and behavioural outcomes such that beliefs about influences were more reflective of current evidence (i.e. TV is detrimental to language development). Changes in per cent of time sitting, standing and stepping, as measured by the *activ*PAL, were minimal, and effect sizes were small.

**Table 4:** Baseline and post-intervention, adjusted difference and effect sizes for potential mediators outcomes

Outcome variable	Baseline (*M*, 95 % CI)		Post-intervention (*M*, 95 % CI)		Adjusted group mean difference* (*M*, 95 % CI)	Effect Size (Cohen’s *d*)	Bias corrected (Hedges’ *g*)
	Control	Intervention	Control	Intervention			
Amount of time parent believes is acceptable for EM use (mins/day)	81.0 (48.9, 113.1)	84.0 (49.2, 118.8)	66.0 (43.8, 88.2)	70.9 (48.3, 93.5)	4.9 (−21.0, 30.7)	0.17	0.09
Weekly frequency young child watches TV/DVD/videos to settle for bed (days/week)	1.9 (0.0, 3.7)	3.0 (0.7, 5.2)	2.3 (17.6, 4.3)	2.6 (0.5, 4.7)	−0.6 (−2.0, 0.8)	0.39	0.22
Parent feels they can support active opportunities (possible range 0–4)#	3.2 (2.7, 3.7)	3.5 (3.3, 3.7)	3.4 (3.0, 3.8)	3.6 (3.3, 3.8)	−0.1 (−0.3, 0.1)	0.36	0.20
Parent believes it is important to participate in activities together (possible range 0–4)#	1.3 (1.0, 1.7)	1.7 (1.3, 2.1)	1.4 (1.1, 1.7)	1.6 (1.1, 2.1)	0.0 (−0.5, 0.5)	0.05	0.03
Parental self-efficacy to limit child’s EM use (possible range 0–4)#	2.4 (2.0, 2.8)	2.5 (2.0, 3.1)	2.6 (2.2, 3.0)	2.8 (2.4, 3.3)	0.1 (−0.2, 0.5)	0.31	0.17
Parental beliefs about influence of EM use on health, developmental and behavioural outcomes (possible range 0–4)^	2.1 (1.7, 2.4)	2.0 (1.7, 2.4)	2.2 (1.9, 2.5)	2.4 (2.1, 2.8)	0.3 (−0.2, 0.7)	0.55	0.31
Child’s opportunity to participate in EM use (possible range 0–4)~	1.5 (1.3, 1.7)	1.7 (1.5, 2.0)	1.5 (1.3, 1.6)	1.6 (1.4, 1.8)	0.0 (−0.2, 0.2)	0.12	0.07
*activ*PAL™							
Percent of time sitting	54.4 (47.7, 61.2)	54.2 (48.9, 49.5)	54.8 (45.5, 64.0)	54.1 (49.7, 58.5)	1.0 (−7.7, 9.7)	0.11	0.06
Percent of time standing	30.3 (25.0, 35.5)	30.7 (27.0, 34.3)	30.8 (21.5, 40.1)	30.3 (27.8, 32.7)	1.6 (−6.9, 10.1)	0.18	0.10
Percent of time stepping	15.3 (13.2, 17.4)	15.2 (11.8, 18.7)	14.5 (11.0, 17.9)	15.7 (11.8, 19.6)	1.3 (−2.6, 5.3)	0.31	0.18

#higher values indicate more favourable outcomes; ^ higher values indicate parental knowledge consistent with evidence; ~ lower values represent more restricted opportunities

## Discussion

The results show that *Family@play* was feasible, acceptable and potentially efficacious in reducing children’s electronic media use. Although recruitment was initially difficult, recruitment targets were achieved and exceeded. Despite 100 % retention of participants in the control group throughout the program, retention rates in the intervention group were lower, with 50 % of participants withdrawing after randomisation and before the program concluded. The program in the form delivered may be most suitable to families from high SEP backgrounds and whose children participate in higher levels of electronic media use. However, all data were successfully collected for all participants completing the program, showing that the use of TUDs, *activ*PAL™ accelerometers, and comprehensive surveys is feasible within this population. The program was clearly acceptable to parents as evidenced by the high process evaluation ratings and positive feedback.

The alignment of the sessions with Social Cognitive [24] and Family Systems [22] theories may have contributed to the high process evaluation scores. Specifically, the focus of the program on the three components of selectively and non-selectively reducing, and displacing, EM use, likely contributed to such high ratings. Inclusion of age-appropriate strategies and the opportunity for participants to share experiences and ideas was relevant and attractive to participants. Behaviour change programs with similar focus and strategies have previously been efficacious in older children [38].

The results show encouraging effect sizes for the primary outcome of total EM use, with a reduction of 39 mins for the intervention group compared with an increase of 3 mins in the control group. Encouraging effect sizes were also evident for several individual EM behaviours. Sedentary EM use decreased by 36 mins for the intervention group and three mins in the control group. A moderate effect size was evident for DVD/video viewing, a passive pursuit similar to TV viewing [39]. The effect size for TV viewing approached the moderate level, with a 17 % reduction in time spent in that behaviour in the intervention group. The majority of children’s EM use is TV viewing and therefore it is essential that interventions target that behaviour. Similar to the findings in this study, Hip Hop to Health achieved a significant reduction in total screen time (measured as TV/DVD/video viewing, computer, video games) but was unsuccessful in reducing TV viewing time in itself [40]. Possibly this is due to the need many parents report for TV to be used as a babysitter when they need to attend to other responsibilities and to have some ‘time out’ from their children [22, 41]. Other programs have achieved positive results in decreasing EM use when reported as TV and computer use [42] and TV but not computer use [43]. Given the differing nature of participation in each type of EM [39], future studies should report on different types of EM use separately. The bulk of the evidence on the adverse health and developmental outcomes for EM use in young children focuses on TV viewing [44]. Therefore, identifying and testing strategies and activities to reduce this ubiquitous behaviour is essential to support healthy outcomes in children. Additionally, evidence of potential health outcomes from other types of EM use is desperately needed. Ideally, parents require support in implementing activities which keep children quietly and safely engaged while other responsibilities are attended to. Other activities, such as block play, puzzles, and looking at books, have developmental benefits for children and this may be particularly salient for parents [39].

Encouragingly effect sizes approaching or at the moderate level were also seen in several parental variables which may be key mediators of children’s EM use (mediation was not tested due to small sample size) [45]. Specifically, parental perceptions of adverse health outcomes from EM use were improved in line with current evidence during the program in the intervention group. Parental perceptions, beliefs and behaviours have previously been shown to be associated with children’s EM use [46, 47] and it is encouraging that a short intervention such as *Family@play* is able to impact such key variables.

A recent review identified several studies which reported intervention effects on EM use during early childhood and identified one family-based study which included 2–3 year old children, targeting TV and computer use [19, 20]. All of the studies in that review had been conducted in the United States. Since that review, one study has been identified which included EM use during early childhood and was conducted outside the US. That study targeted parents of new-born children until the age of 20 months and reported significant effects on TV viewing at the conclusion of the 15 months program [48] but did not target other EM behaviours. Therefore, *Family@play* is the first study to deliver a family-based intervention targeting parents of two-three year-old children outside the United States and the first family-based intervention in this age group targeting multiple EM behaviours.

Despite an overall reduction of children’s EM use, only small effect sizes were seen for children’s time sitting, standing and stepping. It is possible that decreases in children’s EM use were replaced by other sedentary behaviours such as quiet play, crafts or looking at books. In addition to the strategies included in this study to decrease children’s EM use, future interventions may wish to actively target reductions in sitting time and consider inclusion of strategies which promote active play to decrease children’s sitting time.

Limitations of this study include the small sample size and retention rate in the intervention group. These results are therefore more potent in supporting the feasibility and acceptability of the program. Although participant retention in the control group was good when those participants took part in the program, the high drop-out rate in the intervention group suggests that alternative forms of delivery, such as online delivery of some or all of the program, as suggested by the participants themselves, need to be investigated to further assess feasibility of this program in this population. The study included several strengths. The facilitator trained to deliver the program was not a researcher nor had any particular expertise in the content area. Therefore, this supports the potential generalizability and sustainability of the program. The detailed session plans developed and used by the facilitator provide an important resource for further testing of this program in a larger sample. The use of TUDs, although a proxy-report measure which may be subject to social desirability bias, is important to note as it may provide a more accurate reflection of the time children spend in actual behaviours rather than using a weekly global measure. Such diaries have been shown to have high levels of validity and reliability in adults [49]. Further, this study was a true pilot randomised controlled trial. It therefore provides valuable and essential information to inform a future, larger-scale trial [50]. Despite being low powered as a pilot study, effect sizes in several key outcome variables and other potential mediators are substantial enough to warrant a larger trial with greater statistical power.

## Conclusion

The *Family@play* program was successful in decreasing total EM use. Future studies should specifically measure different types of EM use as participation differs, as do strategies which may be effective in changing specific behaviours. Programs seeking to target reductions in sitting time should include strategies specific to that behaviour.

## Abbreviations

EM: electronic media
FST: family systems theory
ITT: intention to treat
SCT: Social cognitive theory
TUD: time use diary
